# Contrast-Free FLIM Reveals Metabolic Changes in Pathological Islets of Langerhans

**DOI:** 10.3390/ijms232213728

**Published:** 2022-11-08

**Authors:** Polina Ermakova, Alena Kashirina, Irina Kornilova, Aleksandra Bogomolova, Darya Myalik, Nasipbek Naraliev, Denis Kuchin, Liya Lugovaya, Elena Zagaynova, Vladimir Zagainov, Aleksandra Kashina

**Affiliations:** 1 Institute of Experimental Oncology and Biomedical Technologies, Privolzhsky Research Medical University, 10/1 Minin and Pozharsky Pl., 603005 Nizhny Novgorod, Russia; 2 Nizhny Novgorod Regional Clinical Hospital Named after N.A. Semashko, 190 Rodionova Str., 603126 Nizhny Novgorod, Russia; 3 Institute of Biology and Biomedicine, Lobachevsky State University of Nizhny Novgorod, 23 Gagarin Ave., 603022 Nizhny Novgorod, Russia; 4 State Budgetary Healthcare Institution “Nizhny Novgorod Regional Clinical Oncological Dispensary”, 11/1 Delovaya Str., 603163 Nizhny Novgorod, Russia

**Keywords:** islets cells, diabetes, insulin-deficient conditions, fluorescence lifetimes, FLIM, NAD(P)H

## Abstract

FLIM (Fluorescence Lifetime Imaging Microscopy) is a powerful tool that could be used in the future to diagnose islet cell recovery after therapy. The identification of appropriate FLIM parameters is required to determine islet quality and islet cell metabolism throughout the organ under various conditions of insulin deficiency. The aim of the work was to identify key FLIM parameters, changes of which are characteristic of pancreatic pathologies. The τm, τ1, τ2, α1, α2 and α1/α2 of free and bound forms of NAD(P)H of the islet cells of animals (rats and pigs) and of humans with and without pathologies were measured and analyzed. The data were confirmed by IHC and histological studies. We identified three FLIM parameters in islet cells from animals with streptozotocin (STZ)-induced diabetes mellitus (DM) and from humans with chronic pancreatitis + type 2 diabetes (T2D), which differ in the same way: τm and α2 take lower values compared to the nonpathological islet cells, while α1/α2 takes higher values. In islet cells from patients with adenocarcinoma (PDAC) and chronic pancreatitis, these parameters had reverse tendency relative to the norm or did not differ. Thus, minimally invasive and non-contrast FLIM methods may, in the future, be used to diagnose pathological islet cells.

## 1. Introduction

Insulin-deficient conditions such as type 1 and type 2 DM, and pancreatogenic diabetes represent a major global public health problem [1,2]. In T1D (type 1 diabetes), β-cell stress is mediated by autoimmunity however the cause of β-cell stress and dysfunction in T2D is not yet fully resolved, but protein misfolding and the formation of toxic oligomers of the islet amyloid polypeptide (IAPP) are emerging as important contributors [3,4]. After a critical decrease in the mass of the pancreatic parenchyma, pancreatogenic diabetes mellitus (PDM) develops, which differs from T1D. The reasons for such a loss of the critical mass of islets can be total pancreatectomy, pancreatic necrosis, chronic fibrosing diseases, atrophy of the pancreas due to chronic inflammation, and tumors [5,6].

Β-cell dysfunction is, at least in part, due to remodeled glucose metabolism as a component of a conserved pro-survival signaling program. In healthy β cells, as glucose concentrations increase, there is a proportionate increase in adenosine triphosphate (ATP) generation from oxidative phosphorylation (OXPHOS) that, in turn, acts on membrane K_ATP_ channels, electrically coupling oxidative phosphorylation to insulin secretion [7]. It has been suggested that there is increased glycolysis in stressed β cells that is largely unrelated to oxidative phosphorylation in both T1D and T2D, but is consistent with this kind of high survival-inducing Warburg level characteristic of cancer cells [8].

To date, most studies of cell metabolism are based on methods such as PCR (polymerase chain reaction), immunocytochemistry and transcriptomic studies, which, on the one hand, do not give a complete picture of the islets in native tissue, and, on the other hand, are quite contradictory. Along with traditional methods, optical imaging methods are increasingly being used to solve biomedical problems, among which FLIM is the most promising. FLIM based on recording the average time that a fluorophore remains in an excited state before emitting a photon (fluorescence lifetime). It is important to note that the fluorescence lifetime is determined by the chemical structure of the fluorophore and its microenvironment and does not depend on the concentration of the fluorophore and the intensity of laser radiation. In previous studies, scientists have already successfully applied FLIM to metabolic investigations of various cell models [9,10,11,12,13,14]. It is a modern, highly sensitive method for the analysis of cellular metabolism by recording the intrinsic (auto-) fluorescence of the metabolic cofactors reduced NAD(P)H (nicotinamide adenine dinucleotide (phosphate)) and oxidized FAD (flavin adenine dinucleotide). The advantages of this approach are the absence of the need for sample staining, the possibility of direct visualization of the metabolic status, and the high molecular specificity of the method. Pathological β cells differ in their metabolism and biochemical profiles from healthy cells this being reflected in the characteristics of the fluorescence decay of the cofactors NAD(P)H and FAD, the different states of which (free or bound to protein, closed or open conformation) are involved in different metabolic pathways and redox reactions within the cells. As with tumor cells, diabetic β cells have a more glycolytic metabolism that is manifested in an increase in the fraction of the free form of NAD(P)H and a weak intensity of FAD fluorescence. Using a mouse model of T2D and human islets from organ donors with and without T2D, and combining FLIM and confocal imaging has illustrated the heterogeneity of the β and α cell responses to increased glucose concentration in the responses of intact islets cells, and in the metabolic trajectory in the setting of β-cell stress [15]. Using the same approach Zbinden A. et al. assessed pseudo-islet quality and their glucose-response under standard and hypoxic conditions [16]. A group headed by Piston D.W. using only two-photon excitation microscopy compared the glucose-induced NAD(P)H autofluorescence response in dispersed β cells and of such cells within intact islets, revealing the heterogeneity of responses in the dispersed cells compared to cells contained within islets [17]. They also detected a glucose-stimulated increase of NAD(P)H fluorescence in intact pancreatic islets, with most of the glucose-induced increase in NAD(P)H corresponding to the location of the mitochondrial network [18].

Thus, metabolic FLIM represents a powerful tool that may also have the potential to be used for diagnostics in treatments aimed at restoring islet cell function in diabetes. To bring FLIM closer to clinical reality, new FLIM criteria are needed to identify islet quality and metabolism in the whole organ under various insulin-deficient conditions.

In our work, we extended the capabilities of FLIM to assess the metabolism of islet cells in the pancreas of the pig and rat (with and without streptozotocin (STZ)-induced DM) and in the human pancreas (with chronic pancreatitis, chronic pancreatitis + T2D and PDAC).

## 2. Results

### 2.1. Live Cell Imaging of Islets in Whole Pancreas from Rat Models of Type 1 Diabetes

Type 1 DM results from autoimmune destruction of β cells, with the development of absolute insulin deficiency. The STZ model for the development of type 1 diabetes mellitus is a common animal model. The STZ selectively enters pancreatic beta cells via the GLUT2 glucose transporter where it causes damage to the genetic machinery and ultimately leads to beta cell death [19,20].

The glycolysis becomes the predominant means of obtaining energy in the β cells that are being destroyed during the development of STZ-induced diabetes. The contribution of the main metabolic pathways (glycolysis and OXPHOS) to the total energy metabolism of islets cells from both STZ-induced DM and healthy animals was assessed by analysis of the fluorescence lifetimes (τm, τ1, τ2) and the fluorescence lifetime contributions (α2 and α1/α2) of the free and bound forms of NAD(P)H, using FLIM.

STZ injection rapidly produced the characteristic signs of diabetes such as increased intake of the water, weight lost and increased blood glucose concentrations. The average weight of the rats decreased from 222 ± 8 g to 158 ± 10 g, accounting for 23% of the original weight. The mean blood glucose level in rats with diabetes induced by a dose of STZ of 65 mg/kg was 27.0 ± 8.5 mmol/L and persisted for 3 months. A glucose tolerance test showed that in rats with STZ-induced DM, the level of glucose in the blood did not undergo any increase in the first 20 min after the administration of 40% glucose, while in healthy rats there was an increase by an average of 6–13 mmol/L. An hour later, while in healthy animals, the glucose level had then fallen by 10 mmol/L, in animals with STZ-induced DM, the glucose level either did not decrease or decreased by only 2–3 mmol/L.

**Figure 1:**
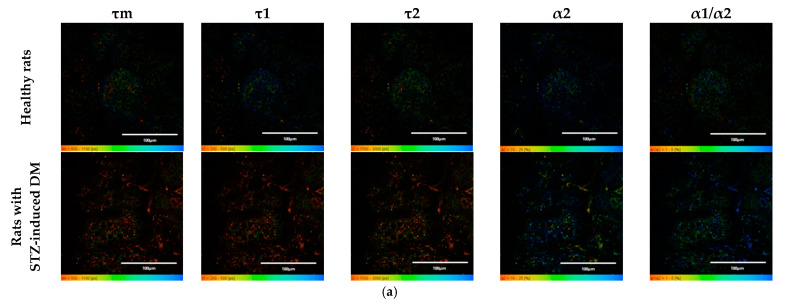

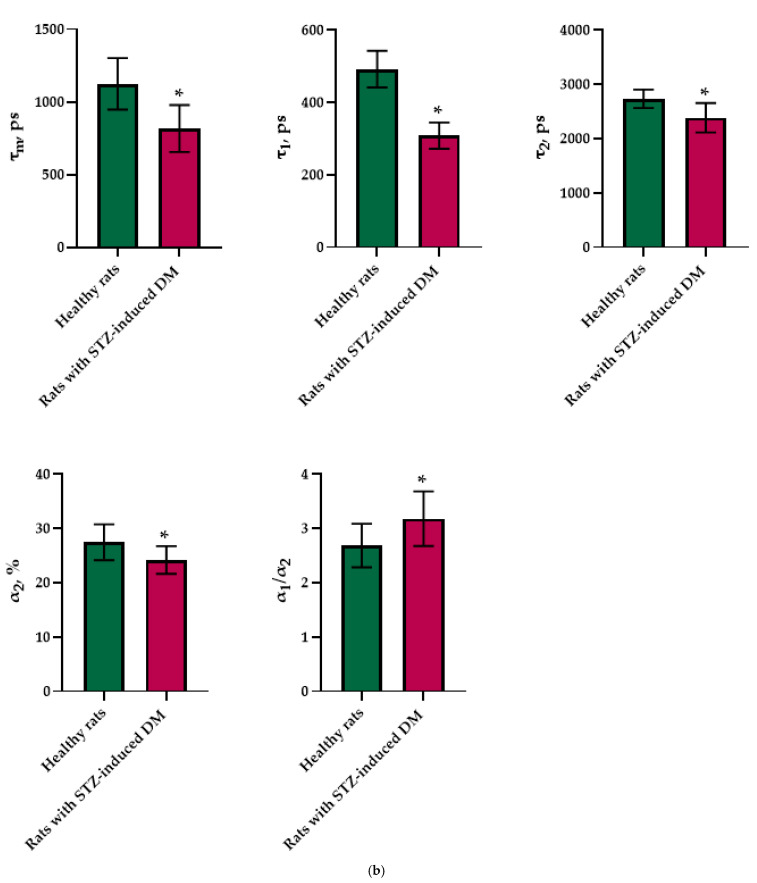
FLIM of NAD(P)H in pancreatic islets of healthy rats and rats with STZ-induced DM. (**a**) Pseudo-color-coded images of τm, τ1, τ2, α2 and α1/α2 of the NAD(P)H in pancreatic islets of healthy rats and rats with STZ-induced DM (scale bars: 600–1100 ps (τm), 200–500 ps (τ1), 1500–3000 ps (τ2), 10–25% (α2) and 1–5 (α1/α2)). The scale length in all pictures is 100 µm. (**b**) Mean values of τm, τ1, τ2, α2 and α1/α2 of the NAD(P)H in pancreatic islets of healthy rats and rats with STZ-induced DM (mean ± SD). *—*p* = 0.00000 (τm, τ1, τ2) and *p* = 0.001 (α2, α1/α2).

An analysis of the FLIM data showed that the islets cells of rats with STZ-induced DM differed from those of healthy rats in all tested parameters (*p* ≤ 0.05). Namely, the islets cells of rats with STZ-induced DM were characterized by lower values of τm, τ1, τ2, and α2 and higher values of the fluorescence lifetime contributions ratio (α1/α2) compared to the islets cells of healthy rats (Figure 1). Despite the fact that each of the parameters of islet cells of rats with STZ-induced DM is statistically significantly different from cells without pathology, several parameters can be distinguished, for which the differences between them is particularly pronounced. These are the parameters τm and τ1. Moreover, based on the values of α2 and α1/α2, we can assume that glycolysis predominates in the islets cells of rats with STZ-induced DM, while the islets cells of rats without pathology rely predominantly on OXPHOS.

**Figure 2:**
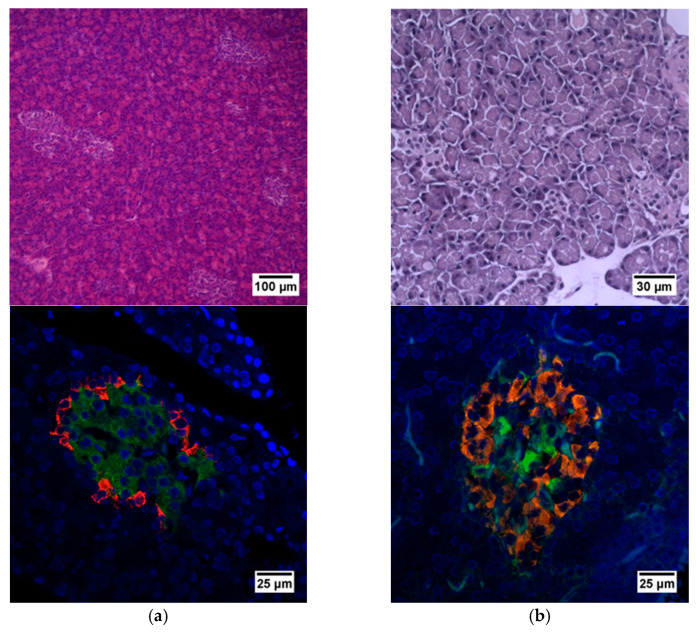
Histological and immunohistochemical analysis of rat pancreas. (**a**) An islet of Langerhans of a healthy rat, and (**b**) an islet of Langerhans of a rat with STZ-induced DM. Staining with hematoxylin and eosin. Staining with antibodies to glucagon (red) and to insulin (green) and nuclei (blue).

**Figure 3:**
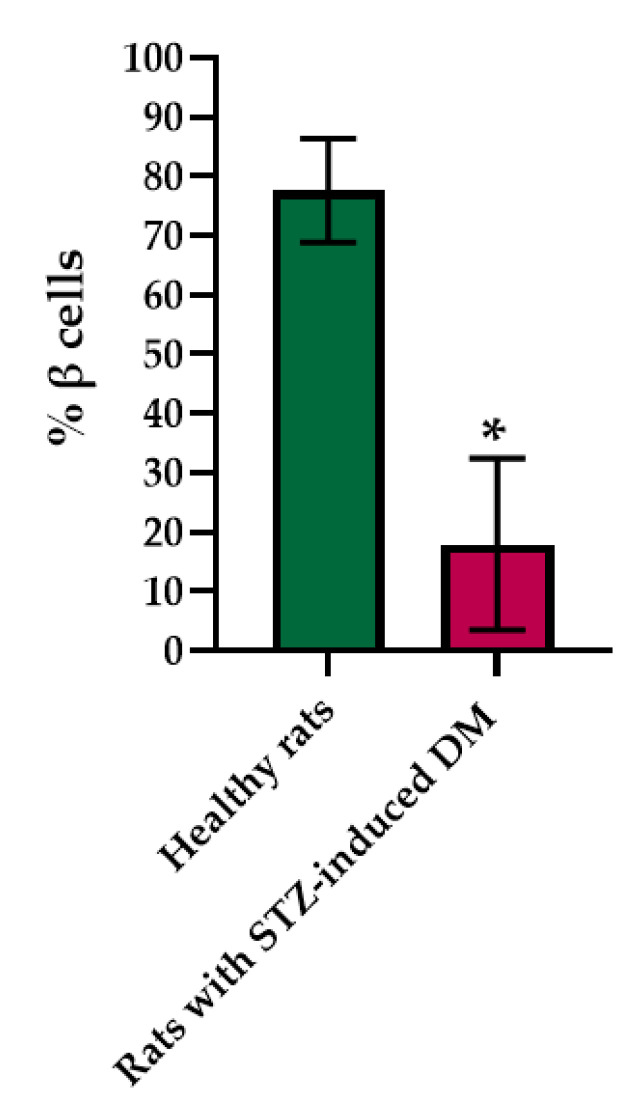
Analysis of the percentage of beta cells in the pancreas islets of healthy rats and of rats with STZ-induced DM. *—*p* = 0.00000.

Histological staining of pancreatic samples with hematoxylin and eosin showed a decrease in the size of the islets of diabetic rats compared to healthy animals (Figure 2). It was also found that the nuclei of the islets of animals with STZ-induced DM were larger relative to the norm. The islet nuclei of diabetic rats have different chromatin content. Islet cells in diabetic rats were mostly round or oval, but irregularly shaped cells were also found. Some of the cells were markedly vacuolated. The capsule of the islets was located discretely with gaps. Membrane-destroyed cells were present. Fibrosis of the exocrine part could be observed. There was visible vascular thrombosis. The cells of the islets of normal animals did not show these effects. In these, the cell nuclei were rounded, and all cells had a weakly basophilic cytoplasm, which indicated an intensive metabolism. They also displayed a moderate plethora of blood vessels, a normal blood supply and no hemorrhages. Immunohistochemical analysis showed (Figure 2) that, in the islets of healthy rats, β cells producing insulin were located in the center, while the α cells synthesizing glucagon were located along the periphery. In islets of healthy rats, on average, 77.6% of the area was occupied by beta cells. In diabetic animals, beta cells comprised only 17.9% of the islet area (Figure 3).

Therefore, the morphological and immunohistochemical results correlate well with the results obtained by FLIM and confirm the predominance of the contribution of glycolysis to the overall metabolism of the pathological islets cells.

### 2.2. Live Cell Imaging of Islets in Whole Pancreas from Pig Models with STZ-Induced DM

The day after the STZ injection, the pigs appeared weak, and one animal was vomiting. Additionally, in the first days after induction, a characteristic loss of appetite was observed. After, the start of insulin therapy, the state of health returned to normal. The pigs lost an average of 1 kg in weight before starting insulin therapy, during which they began gradually to increase their weight. The mean blood glucose level in the pigs during the first 7 days was 15.3 ± 4.6 mmol/L, and during insulin therapy, it dropped to 9.4 ± 3.4 mmol/L in the morning and 10.8 ± 3.2 mmol/L in the evening for 3 months. Diabetes was confirmed by a glucose tolerance test 7 days after induction and every month thereafter. The level of glucose in the blood of healthy pigs and of pigs with STZ-induced DM in the first 20 min after the injection of 0.5 g/kg of glucose increased by 3–5 mmol/L. After that, in normal animals, the level of glucose in the blood decreased and completely returned to normal after 2 h. In the diabetic pigs, after one hour, the glucose level had not fallen, and after 2 h, had either remained constant or dropped by only 1–1.5 mmol/L.

**Figure 4:**
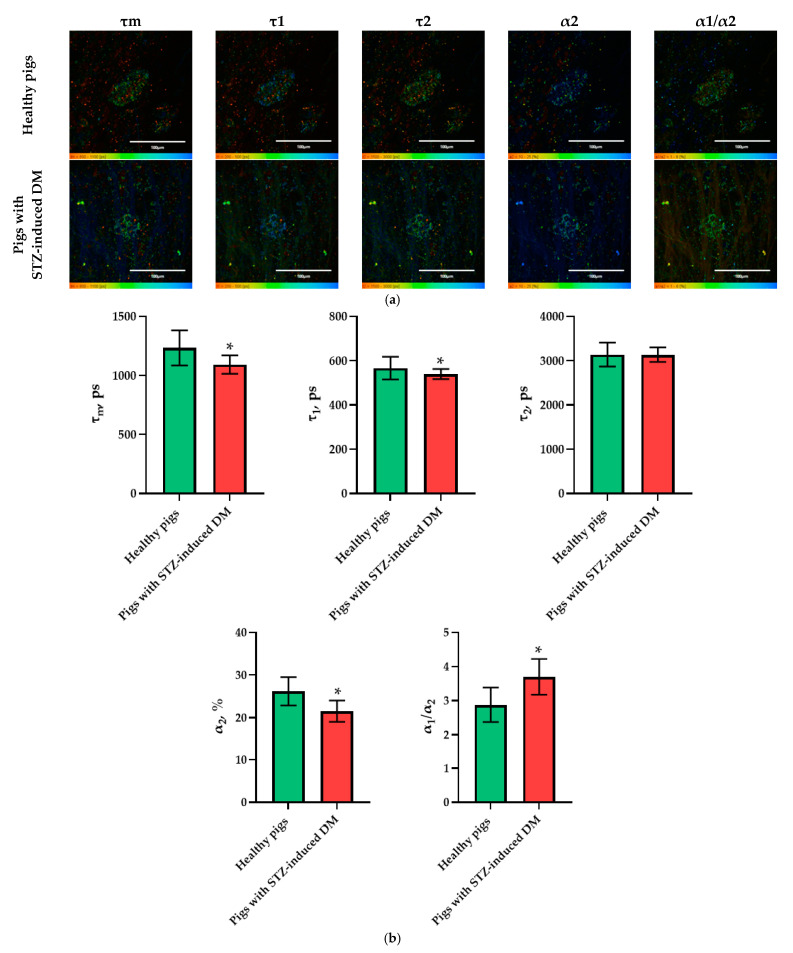
FLIM of NAD(P)H in pancreatic islets of healthy pigs and of pigs with STZ-induced DM. (**a**) Pseudo-color-coded images of τm, τ1, τ2, α2 and α1/α2 of the NAD(P)H in pancreatic islets of healthy pigs and in pigs with STZ-induced DM (scale bars: 600–1100 ps (τm), 200–500 ps (τ1), 1500–3000 ps (τ2), 10–25% (α2) and 1–6 (α1/α2)). The scale length in all pictures is 100 µm. (**b**) Mean values of τm, τ1, τ2, α2, α1/α2 of the NAD(P)H in pancreatic islets of healthy pigs and in pigs with STZ-induced DM (mean ± SD). *—*p* = 0.00000.

In the pig models, an analysis of the FLIM data revealed statistically significant differences in the following parameters: τm, τ1, α2, and α1/α2. Moreover, in the islet cells of pigs with STZ-induced DM, τm, τ1 and α2 took on lower values, while α1/α2 took on higher values in comparison with the islet cells of pigs without pathology (Figure 4). As with the analysis of data on the rat models, the parameters showing the greatest differences between STZ-induced DM cells and cells without pathology were identified. In the pigs’ islets, these parameters were: τm, α2 and α1/α2.

**Figure 5:**
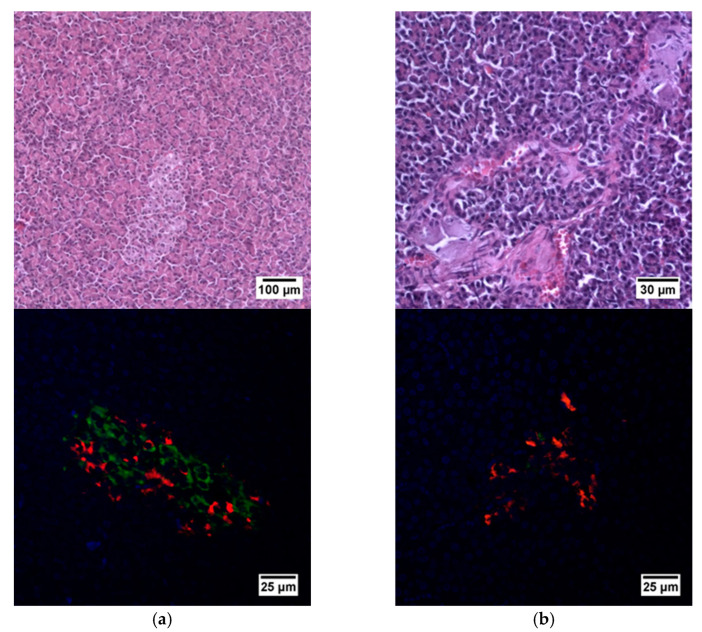
Histological (upper row) and immunohistochemical (lower row) analysis of the pig pancreas. (**a**) An islet of Langerhans of a healthy pig, and (**b**) an islet of Langerhans of a pig with STZ-induced DM. Staining with hematoxylin and eosin. Staining with antibodies to glucagon (red) and to insulin (green) and nuclei (blue).

**Figure 6:**
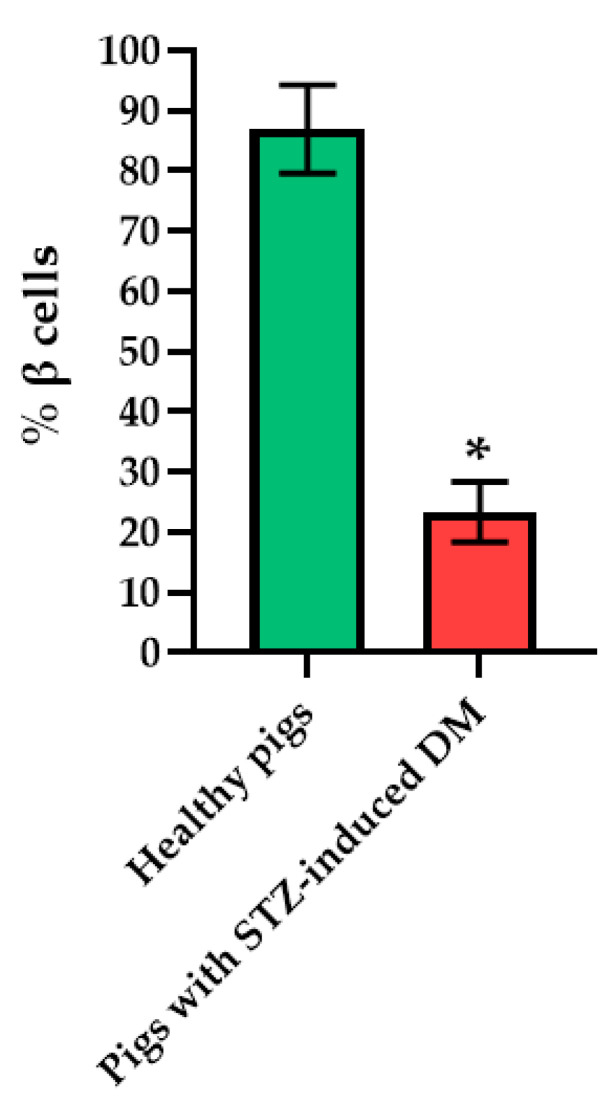
Analysis of the percentage of beta cells in the islets of the pancreas of healthy pig compared with a pig with STZ-induced DM. *—*p* = 0.00000.

Histological analysis of pancreas samples using hematoxylin and eosin staining confirmed the presence of diabetes mellitus in the STZ pigs (Figure 5). When examining the pancreas of a diabetic pig, it was difficult to distinguish between the endocrine and exocrine portions. The structure of the islets was disturbed, there was cell edema (hyperchromic nuclei, coarse-grained cytoplasm). There were also dystrophic changes (sinusoids were empty, open). There were seals of the cytoplasm in some cells. In normal pancreas, the boundaries between the exocrine and endocrine parts were clear, the cytoplasm was weakly basophilic and fine-grained, and the cells were large. On the peripheries of the islets, the cells had rounded nuclei, with a high proportion of finely dispersed chromatin. In the center of the islets, the nuclei were larger, lighter and with a finely dispersed content of chromatin. The sinusoidal capillaries were dilated, there was no edema, all typical of the norm. Using IHC analysis, in the diabetic pigs, a decrease in the size of the islets was noted, due to death of a proportion of insulin-producing β cells, although the glucagon-producing α cells did not appear to have suffered (Figure 6).

Thus, FLIM in combination with histological and IHC analysis indicated the more glycolytic phenotype of the islets in the pathological pig pancreas, as in the case of the diabetic rat islets.

### 2.3. Live Cell Imaging of Islets in Whole Pancreas from Nonpathological and Pathological Human Patients (Chronic Pancreatitis + T2D, Chronic Pancreatitis, PDAC)

After analyzing the energy metabolism of cells of the islets of laboratory animals, we used FLIM to study the metabolism of human islet cells from nonpathological patient and from patients with three types of pathological conditions (Table 1).

**Figure 7:**
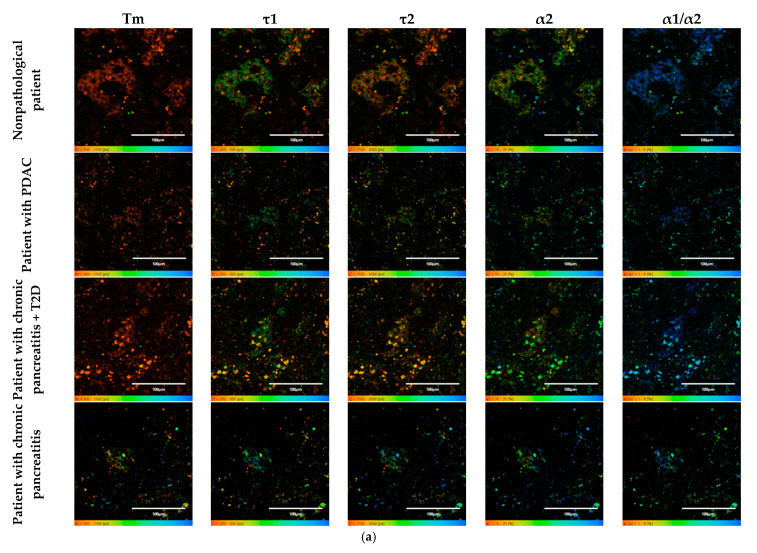

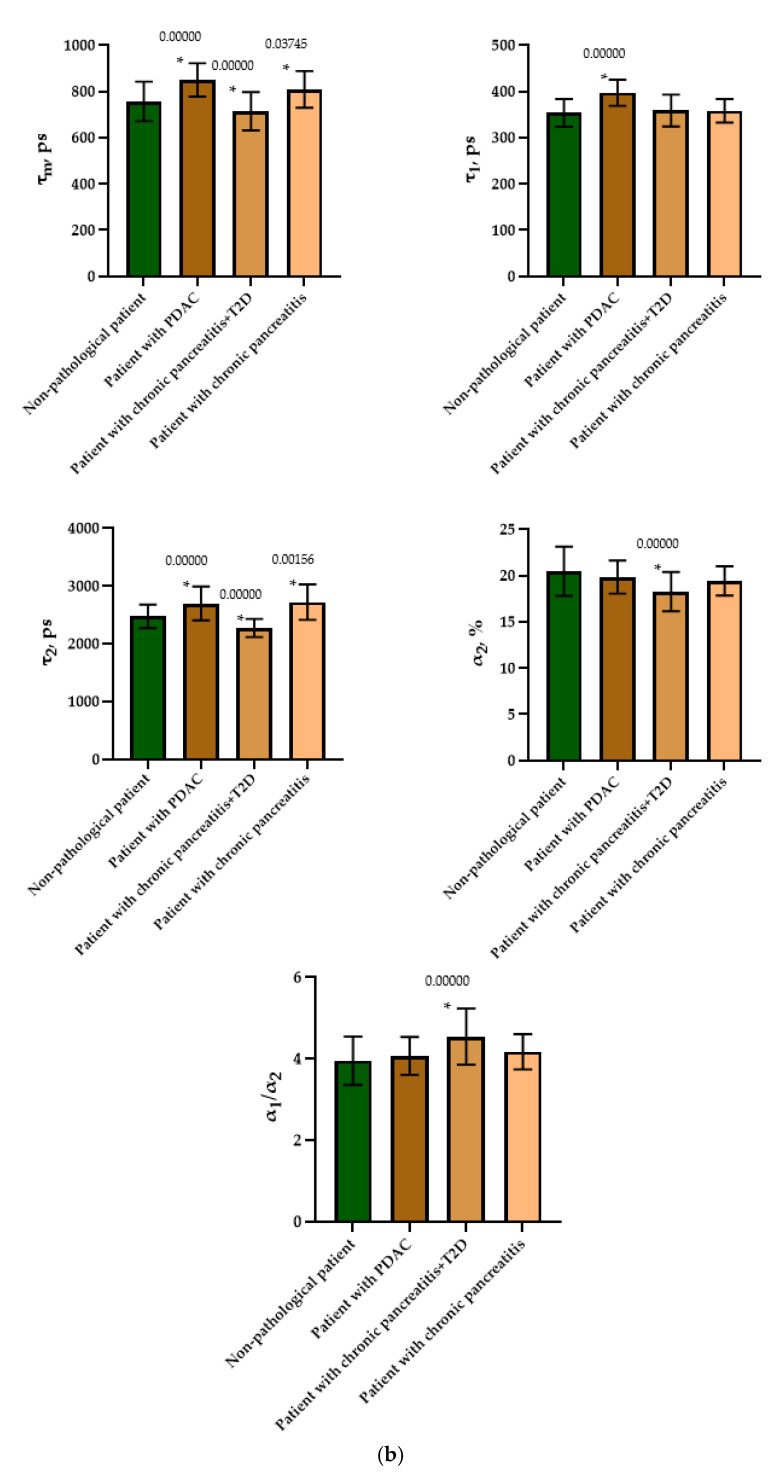
FLIM of NAD(P)H in human islets of nonpathological patient and islets with pathologies (PDAC, chronic pancreatitis + T2D and chronic pancreatitis). (**a**) Pseudo-color-coded images of τm, τ1, τ2, α2 and α1/α2 of the NAD(P)H in human islets of nonpathological patient and islets with pathologies (scale bars: 600–1100 ps (τm), 200–500 ps (τ1), 1500–3000 ps (τ2), 10–25% (α2) and 1–6 (α1/α2)). The scale length in all pictures is 100 µm. (**b**) Mean values of τm, τ1, τ2, α2 and α1/α2 of the NAD(P)H in human islets of nonpathological patient and islets with pathologies (mean ± SD). *—*p* ≤ 0.05.

To do this, as in the case of the animal pancreas samples, the following parameters were analyzed: fluorescence lifetimes (τm, τ1, τ2) and the fluorescence lifetime contributions (α2 and α1/α2 ratio) of the free and bound forms of NAD(P)H. When comparing the parameters of the islet cells of nonpathological patients and of a patient with PDAC, a statistically significant difference was revealed in three parameters, namely, the fluorescence lifetimes (τm, τ1 and τ2), with the highest values being in the cells of the patient with pathology. No differences were found in the contribution of the bound form of NAD(P)H nor in the ratio of the contributions of the free and bound forms of the coenzyme, which may indicate that the two subjects have similar energy metabolism. When comparing the islet cell parameters of nonpathological patient and a patient with chronic pancreatitis + T2D, statistically significant differences were found in 4 FLIM parameters out of the 5 studied (τm, τ2, α1, α2 and α1/α2) (Figure 7). It is important to note that in the islet cells of the patient with chronic pancreatitis + T2D, the contribution of the bound form of NAD(P)H was lower than in the cells from the nonpathological patient, which may indicate the former’s more glycolytic phenotype. Additionally, the result is consistent with the data on laboratory animals: the islet cells of animals with STZ-induced DM also had a more glycolytic phenotype compared to cells of healthy animals. When comparing the parameters of the islet cells of nonpathological patient and a patient with chronic pancreatitis, a statistically significant difference was found in only two out of the five parameters, namely, the average fluorescence lifetime and the fluorescence lifetime of the bound form of NAD(P)H were both greater in the cells of the patient with this pathology.

Thus, based on the obtained results, it can be established that τm and τ2 are FLIM parameters, that make it possible to distinguish two of the pathological conditions from the nonpathological. It is important to note that the islet cells of the patient with chronic pancreatitis + T2D differed in the fluorescence lifetime contributions of bound NAD(P)H (α2) and the ratio of the fluorescence lifetime contributions (α1/α2) from the cells of nonpathological patient, correlating well with the results obtained from animals with STZ-induced DM.

**Figure 8:**
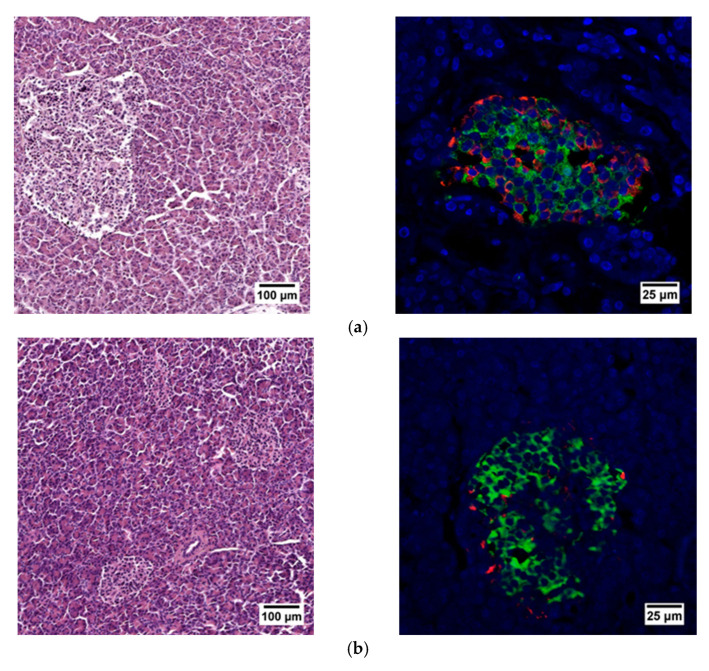

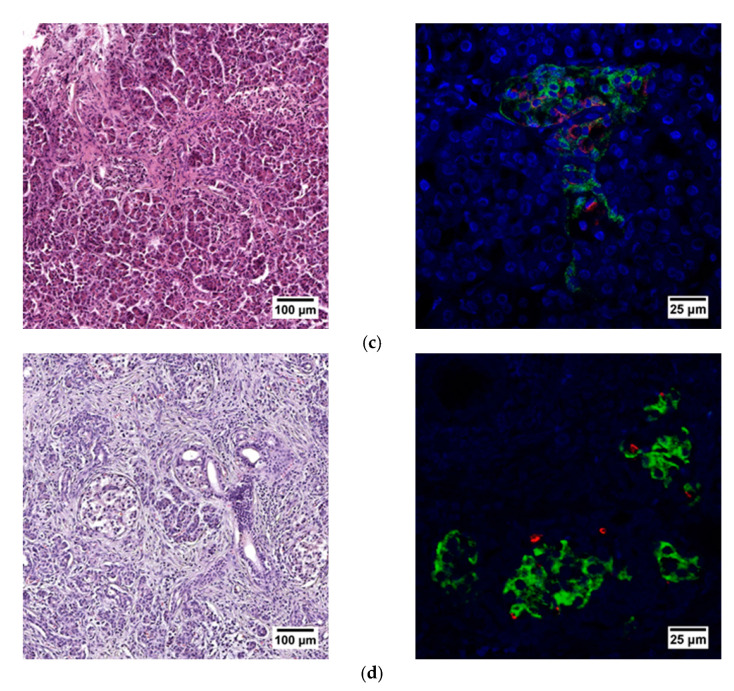
Histological (left) and immunohistochemical (right) analysis of the human pancreas. (**a**) nonpathological patient; (**b**) patient with PDAC; (**c**) patient with chronic pancreatitis + T2D and (**d**) patient with chronic pancreatitis.

**Figure 9:**
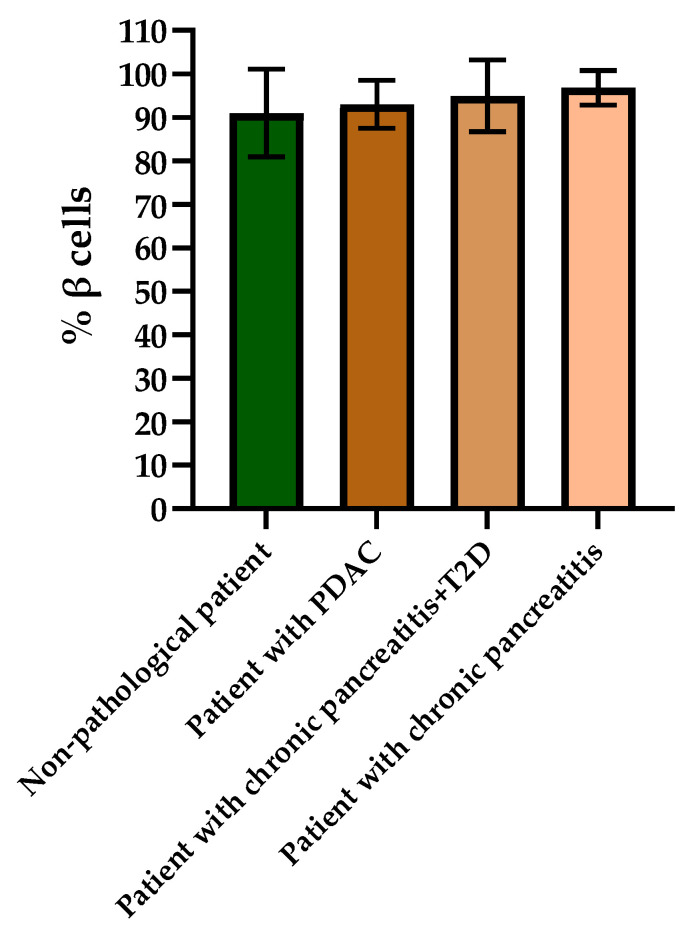
Analysis of the percentages of beta cells in the islets of each type of pancreas.

When evaluating the histological picture, we found that the pancreatic tissue of the nonpathological patient had a lobular structure with the inclusion of mature adipose tissue. The parenchyma was represented by acinar glands and by a typically compact secretory apparatus. The ducts had a typical appearance. In the patient with PDAC the tissue of the pancreas was also lobular in structure, the parenchyma was represented by typical acinar glands and characteristically compact secretory apparatus and ducts were present. The patient with chronic pancreatitis + T2D had pancreatic tissue of lobular structure but with unexpressed fibrosis of the perilobular and intralobular stroma, the secretory apparatus was preserved and represented by compact islets of Langerhans of typical appearance and with typical ducts. In the patient with pancreatitis, deformation of the lobular structures of the pancreatic parenchyma and pronounced fibrosis of the perilobular and intralobular stroma was observed, although the secretory apparatus was preserved and represented by compact islets of Langerhans of a typical type, with intralobular ducts also characterized as being typical. Using IHC analysis we showed that the patients with the different pancreas pathologies did not differ in the ratio of alpha and beta cells (Figure 8 and Figure 9). The obtained results are consistent with the FLIM data and indicate that the islets in patients with PDAC and chronic pancreatitis are similar to nonpathological islets and are characterized by a similar metabolic status. However, the islet cells of the patient with chronic pancreatitis + T2D, despite the absence of serious morphological changes, were characterized by a more glycolytic metabolism, which is apparently associated with the course of type 2 diabetes.

## 3. Discussion

In this work we have conducted the first comparative analysis of the metabolic parameters of islet cells in native pancreas samples of animals (rats and pigs) and humans without pathology and with various insulin-deficient states in order to identify key FLIM parameters, the differences in which are characteristic of pancreatic pathologies.

Stress and dysfunction of the islet cells during the development of pancreatic diseases directly affect their energy metabolism, namely, they are accompanied by a redistribution of the glucose metabolic pathways. It has previously been shown that, in healthy β cells, an increase in glucose concentration contributes to a proportional increase in ATP levels in the cells as a result of OXPHOS, while, in β cells with pathology, increased glycolysis is observed [15,21]. Thus, such features of cell energy metabolism can be used as indicators for diagnostic purposes. To date, the isolation of a sufficient quantity of high–quality islets from the pancreas has been a prerequisite for successful islet transplantation. However, the problem of assessing the quality of the islets both in the whole tissue and in isolated islets without destroying the cell and tissue structure or by using exogenous dyes had not previously been solved. The use of metabolic imaging technologies based on FLIM and autofluorescence of the intracellular metabolites NAD(P)H opens up wide possibilities for minimally invasive and non-contrast diagnostics of the pancreatic islet quality and for determining the number of viable and metabolically active islets.

Wang Z. et al., by using phasor FLIM to analyze the ratio of the fluorescence lifetimes of bound NAD(P)H to the total fluorescence lifetime of the coenzyme, showed that glucose stimulation has a different effect on the energy metabolism, namely glycolysis and OXPHOS, of β and α cells with type 2 diabetes and those without pathology [15]. Additionally, using phasor FLIM but with only one FLIM parameter, namely the ratio of the fluorescence lifetime of bound NAD(P)H to the total fluorescence lifetime, Gregg and colleagues studied the effect of age on human and mouse β cell metabolism. The scientists used FLIM to detect reduced islet mitochondrial function in mice over 24 months of age [22]. In a study by Ferri and colleagues, changes in the metabolism of Insulinoma 1E cells in their under-response to glucose stimulation were analyzed by evaluating the change in the fluorescence lifetime ratio of the bound to the free form of NAD(P)H using phasor FLIM. INS-1E cells were shown to respond to standard glucose stimulation by increasing the bound/free NAD(P)H ratio. However, since non-secreting cells were used as the object of the study, no obvious metabolic shift was found when the cells were stimulated with glucose [23]. Thus, FLIM has proven itself as a minimally invasive method for the analysis of pancreatic islet cells and their analogs. However. for the potential use of FLIM in clinical situations for the assessment of islets before transplantation, more information was required on the FLIM parameters characterizing the state of the islets.

Thus, we analyzed the FLIM parameters of NAD(P)H (τm, τ1, τ2, α2 and α1/α2) in the islet cells of rats, pigs and humans (without requiring separation of the cell types) of whole pancreas. We showed that, in the islet cells of DM animals and in the islet cells of chronic pancreatitis + T2D patient, three FLIM parameters differed from those of nonpathological islets in corresponding ways. Namely, the average fluorescence lifetime of NAD(P)H, and the fluorescence lifetime contributions of the bound form of NAD(P)H take lower values compared to the nonpathological conditions, while the ratio of the fluorescence lifetime contribution of the free form of NAD(P)H to the bound form, on the contrary, takes higher values in comparison with the nonpathological condition. However, in patients with PDAC and chronic pancreatitis, these parameters showed a reverse trend or did not differ. Most likely, in these patients, the inflammatory and oncological processes did not affect the analyzed islets, so they showed a normal metabolic phenotype. Comparing the results of the FLIM analyses with histological and IHC studies of the islet cells of animals in normal and pathological conditions, we assumed that the differences in NAD(P)H fluorescence lifetimes and in the fluorescence lifetime contributions of the free and bound coenzyme forms between healthy and pathological islet cells of the animals were associated as much with a change in the ratio of β and α cells in the islets, due to a decrease in the number of β cells, as with the state of the β cells themselves. Namely, islet cells in a STZ-induced DM pancreas had a different shape, plus different chromatin content was observed in the nuclei, and the cell cytoplasm was vascularized. In patients with PDAC, chronic pancreatitis or chronic pancreatitis + T2D we did not observe any changes in the number of beta cells. Furthermore, according to the IHC analysis, as well as observations, to identify morphological abnormalities by histological examination the islet cells had a typical appearance. Our results obtained using FLIM fully correlated with the described features. Namely, according to the more significant parameters (τm, α2 and α1/α2), there were either no metabolic changes in the cells, or a reverse trend was observed in pathological samples in chronic pancreatitis and PDAC compared to the norm. However, it is worth noting that despite the absence of histological and IHC changes in the islet cells of patients with chronic pancreatitis + T2D, metabolic changes (decreased α2 and increased α1/α2) were found. In these patients, such changes may be associated with the combined effect of pathological acinar tissue and T2D, which to a greater extent, causes metabolic redirection of the islets towards glycolysis.

Indeed, the predominance of glycolysis in diabetic islet cells has previously been shown using non-optical assays. Using transcriptomics and proteomics techniques, significant dysregulation of major metabolic pathways was found in the islets of βV59M diabetic mice. Many genes/proteins involved in glycolysis/gluconeogenesis are up-regulated, while those involved in oxidative phosphorylation are down-regulated [24]. In another study, using Western blotting, it was shown that, in human islets with type 1 diabetes, *GCK* and *HK1/2* are activated, facilitating the flow of glucose through glycolysis. Moreover, the rate of conversion of fructose-6-phosphate to fructose-1,6-bisphosphate is increased in type 1 diabetes by activating *PFKFB3*, increasing pyruvate production [25].

Thus, we identified significant indicative FLIM parameters of NAD(P)H in human and animal islet cells in normal and various pathological conditions, which will further help to advance the FLIM method in studies aimed at assessing the quality of islet cells without the use of exogenous dyes and markers and in a minimally invasive manner.

## 4. Materials and Methods

All our studies with pancreas samples from humans and experimental animals were approved by the local ethics committee of PIMU (protocol No. 10 dated 26 June 2020).

### 4.1. Animal Models and Diabetes Mellitus Induction

Male 8–12-week-old Wister rats, weighing 200– 230 g, and 7–8-month-old female Wisenau pigs, weighing from 13 to 20 kg were used. The rats were bred and housed in pairs under standard environmental conditions with a 12-h light–dark (LD) cycle and habituated to routine handling and restraint. The pigs were housed individually under standardized conditions (19–23 °C; 12:12 h day/night cycle).

The diabetes induction was after a 12 h fast for both rats and pigs. The animals were injected with streptozotocin (STZ) that had been freshly dissolved in citrate buffer (pH 4.5) at a dose of 65 mg/kg of the body weight intravenously for the rats and 150 mg/kg intravenously for the pigs. In the case of the pigs, 24-h glucose monitoring was performed after the drug administration, with measurements every 2 h. When the blood glucose level dropped below 2.5 mmol/L, 0.5 g/kg of glucose were administered. In the case of the rats, 5% sucrose was added to the water.

Diabetes mellitus was confirmed in both the rats and pigs by measurements showing blood glucose concentrations greater than 16.7 mmol/L using a glucometer (AccuChek Active; Roche Diabetes Care, Bern, Switzerland). Animals that failed to develop diabetes were excluded from the study. The experiment lasted 3 months. Control measurements of blood glucose levels were performed every 2 days for the rats and 2 times a day for the pigs to verify diabetes during the entire duration of the experiment. Starting from the 7th day after the administration of STZ, the pigs were injected with insulin NovoMix^®^ 30 FlexPen^®^ (Novo Nordisk, Bagsværd, Denmark) in the morning 10.5 ± 3 U, and 9.6 ± 3 U in the evening, depending on the blood sugar level.

Additionally, the successful induction of diabetes was confirmed by means of a glucose tolerance test. For this a 40% glucose solution was injected intravenously at a dose of 0.5 g/kg. Blood was taken for analysis of glucose level at time points 0, 30, 60 and 120 min.

For FLIM analysis the pancreases of the healthy animals and animals with diabetes (rats, pigs) were removed using rapid surgical procurement (<10 min) and placed in organ preservation solution (10% BSA solution), the cold ischemia time being limited to <40 min. All animal procedures including monitoring, surgery and euthanasia were performed with approval from the local ethics committee of the PIMU. All efforts were made to reduce the quantity of animals required for this study. The rats were anesthetized with Zoletil (Virbac, Hamilton, New Zealand) 6 mg/kg and xylazine (Bayer, Leverkusen, Germany) 90 mg/kg. Premedication of the pigs was carried out with Zoletil 10 mg/kg and xylazine 4 mg/kg, followed by Propofol-Lipuro (B. Braun, Melsungen, Germany) 16 mg/kg/h.

After animals were withdrawn from the experiment the induction of diabetes was confirmed by histological analysis (staining for hematoxylin and eosin) and immunohistochemical analysis (staining for insulin and glucagon) of the pancreases from both the rats and pigs.

### 4.2. Human Pancreas

Human pancreas biopsies were obtained after partial or extensive resections of the pancreas from informed and consenting patients from the State Budgetary Healthcare Institution Nizhny Novgorod Regional Clinical Oncological Dispensary. The characteristics of the pancreas donors are listed in the Results section. Pancreas samples were transported in Custadiol or 5% BSA solution at +40 °C. Imaging was performed 30 min–1 h after transportation. After the imaging procedure the pancreas samples were analyzed histologically and immunohistochemically.

### 4.3. Histology and Immunohistochemistry

Native pancreas tissues were processed for histological and immunohistochemical analysis. Pieces of the pancreas were fixed in formalin and subsequently embedded in paraffin. Seven-micrometer slices of the pancreas were subjected to standard histological wiring and stained with hematoxylin-eosin to visualize the structure. Four-micrometer slices of the pancreas are susceptible to immunofluorescence staining. Immunofluorescence double staining was performed using a primary antibody of glucagon monoclonal (1:10; Invitrogen, Carlsbad, CA, USA) and insulin monoclonal antibody (1:100; Invitrogen, Carlsbad, CA, USA) overnight at 4 °C. The samples were counterstained with DAPI (1:1000; BioLegend, San Diego, CA, USA) according to the manufacturer’s protocol. The percentage of beta cells was calculated using the ImageJ 1.43u program (NIH, Bethesda, MD, USA), as the ratio of the area of beta cells to the area of the entire islets.

### 4.4. Imaging Strategy

Fluorescence and time-resolved images were obtained using an LSM 880 (Carl Zeiss, Oberkochen, Germany) equipped with a short-pulse femtosecond Ti:Sa laser Mai Tai HP laser (Spectra-Physics, Milpitas, CA, USA) with a pulse repetition rate of 80 MHz, and a duration of 140 ± 20 fs and a FLIM system for time-resolved microscopy (Becker & Hickl GmbH, Berlin, Germany). The fluorescence images of NAD(P)H were obtained with two-photon excitation of fluorescence at a wavelength of 750 nm, the fluorescence being detected in the range of 455–500 nm. To obtain FLIM images, the following parameters were used: image size 512 × 512 pixels, field of view size 213 μm × 213 μm; the minimum number of photons per image pixel was 5000; all studies were carried out under constant conditions (37 °C and 5% CO_2_). Using SPCImage 8.6 software (Becker & Hickl GmbH, Berlin, Germany), we analyzed the FLIM images and registered the following parameters: τ1 (ps)—the short fluorescence lifetime that corresponds to the free forms of NAD(P)H and bound forms of FAD; τ2 (ps)—the long fluorescence lifetime that corresponds to the bound forms of NAD(P)H and the free forms of FAD; α1 (%)—the contribution of the short component to the fluorescence lifetime; α2 (%)—the contribution of the long component to the fluorescence lifetime.

### 4.5. Statistics

In our study, the pancreatic islets were analyzed without separation into cell types. For research of the energy metabolism of the islets cells, the images of 27 islets of nonpathological patient, 19 islets of patient with PDAC, 13 islets of patient with chronic pancreatitis + T2D and 2 islets of patient with chronic pancreatitis were analyzed (at least 10 ROI per islet). The data were presented as the mean ± SD or from *n* independent experiments as indicated in the figure legends. The distribution of all the data was first checked for normality using the Kolmogorov–Smirnov and Shapiro–Wilk tests. Statistical analysis of islet cell data was performed using the Mann–Whitney *U* test.

## 5. Conclusions

In our study, we identified the FLIM parameters, characterizing the cellular metabolism, by which it is possible to distinguish islet cells of animals (rats and pigs) and humans with various insulin-deficient conditions from those without pathology. We showed that, in islet cells from animals with STZ-induced DM and patient with chronic pancreatitis + T2D τm and α2 take lower values compared to the nonpathological islet cells, while α1/α2 takes higher values. In islet cells from patients with PDAC and chronic pancreatitis, these parameters had a reverse trend or did not differ, indicating similar metabolic conditions in the pathological cells to normal cells. The FLIM data correlates well with the histological and IHC results. Thus, the differences in the FLIM parameters, previously identified, between the islet cells of healthy animals and those with pathologies can be associated with a change in the morphology of the β cells and a decrease in their number in the islets. In islet cells from patients with PDAC, chronic pancreatitis and in chronic pancreatitis + T2D we did not observe any changes in the number of β cells according to IHC, nor any were any morphological abnormalities revealed by histological analysis. However, it should be mentioned that in islet cells from patient with chronic pancreatitis + T2D, metabolic changes were found that could be associated with metabolic restructuring of the islets towards glycolysis. Our findings will therefore help to advance the value of the FLIM method to researchers needing to investigate the quality of islet cells in a minimally invasive manner.

## Figures and Tables

**Table 1:** Human islets used for FLIM imaging.

Patient	1	2	3	4
Gender	F	M	M	F
Pathology	Non-diabetic	PDAC	Chronic pancreatitis	Chronic pancreatitis + T2D
Number of islets analyzed	27	19	2	13

## Data Availability

Not applicable.
